# Receptor-mediated transcytosis for brain delivery of therapeutics: receptor classes and criteria

**DOI:** 10.3389/fddev.2024.1360302

**Published:** 2024-03-12

**Authors:** Arsalan S. Haqqani, Kasandra Bélanger, Danica B. Stanimirovic

**Affiliations:** Human Health Therapeutics Research Center, National Research Council Canada, Ottawa, ON, Canada

**Keywords:** blood-brain barrier, receptor-mediated transcytosis, drug delivery, trafficking, RMT receptors

## Abstract

The delivery of therapeutics into the brain is highly limited by the blood-brain barrier (BBB). Although this is essential to protect the brain from potentially harmful material found in the blood, it poses a great challenge for the treatment of diseases affecting the central nervous system (CNS). Substances from the periphery that are required for the function of the brain must rely on active mechanisms of entry. One such physiological pathway is called receptor-mediated transcytosis (RMT). In this process, ligands bind to specific receptors expressed at the luminal membrane of endothelial cells composing the BBB leading to the internalization of the receptor-ligand complex into intracellular vesicles, their trafficking through various intracellular compartments and finally their fusion with the abluminal membrane to release the cargo into the brain. Targeting such RMT receptors for BBB crossing represents an emerging and clinically validated strategy to increase the brain permeability of biologicals. However, the choice of an appropriate receptor is critical to achieve the best selectivity and efficacy of the delivery method. Whereas the majority of work has been focused on transferrin (Tf) receptor (TfR), the search for novel receptors expressed in brain endothelial cells (BECs) that can deliver protein or viral vector cargos across the BBB has yielded several novel targets with diverse molecular/structural properties and biological functions, and mechanisms of transcytosis. In this review, we summarize well-studied RMT pathways, and explore mechanisms engaged in BBB transport by various RMT receptors. We then discuss key criteria that would be desired for an optimal RMT target, based on lessons-learned from studies on TfR and accumulating experimental evidence on emerging RMT receptors and their ligands.

## 1 Introduction

Delivering therapeutics into brain tissue is highly restricted due to the presence of a tightly-sealed layer of endothelial cells in brain microvessels that form the blood-brain barrier (BBB). Specialized receptors that are capable of shuttling from the luminal to abluminal side of the BBB, while carrying cargos with them, have provided avenues to deliver therapeutics into this highly restricted area. The specialized pathway(s) that these receptors are involved in is termed receptor-mediated transcytosis (RMT) and thus receptors are usually called “RMT receptors” (Kadry et al., 2020). Currently, several RMT receptors have been discovered at the BBB and demonstrated to deliver therapeutic cargos into the brain in preclinical studies. These range in their biological functions but are mostly involved in transporting physiological ligands or signals intracellularly and include transporters of iron, lipids and solutes, and receptors of insulin, insulin-like growth factors, neuropeptides, and other proteins. While their involvement in the RMT pathway(s) is one of the most important criteria for a receptor to be able to successfully carry therapeutic cargo from the luminal to abluminal side of the BBB, other criteria, such as receptor extracellular domains accessibility on luminal surface of the BBB, their brain specificity/abundance and sequence conservation, are just as important when assessing developability for successful brain delivery with minimal side-effects. In this review, we summarize the different steps in the RMT process and examine various criteria that are either attractive or undesirable in some of the currently known RMT receptors. While we acknowledge that some non-receptor mediated pathways (e.g., adsorptive-mediated transcytosis) are also important for drug delivery into the brain, these pathways are beyond the scope of this review and have been reviewed elsewhere (Kadry et al., 2020; Banks, 2023; Zhang, 2023). Furthermore, this review focuses on criteria for the receptors rather than the criteria for the targeting antibodies, the latter having been reviewed by others and us previously (Haqqani et al., 2022; Pardridge, 2023).

## 2 Receptor-mediated transcytosis

RMT is a multi-step transcellular process that usually occurs in polarized cells and is used by naturally-occurring macromolecules to bypass physiological barriers and enter restricted areas throughout the body. Examples of some of these macromolecules include low-density lipoproteins (LDLs), transferrin (Tf), insulin (INS) and INS-like growth factors 1 and 2 (IGF1, IGF2). These ligands bind to their respective receptors on one surface of the polarized cell resulting in receptor-mediated endocytosis (RME) (step 1), followed by their endosomal sorting (step 2), and eventual exocytosis (step 3) at the opposite surface of the cell. The RMT receptors are thus attractive targets to develop targeting antibodies and peptides as macromolecular Trojan horses for delivering therapeutics across the BBB and into the brain. A significant understanding of the various steps involved in the RMT receptor pathways at the BBB have come from studying trafficking of the targeting antibodies [reviewed previously (Haqqani et al., 2022)]. Here the key steps are briefly summarized, namely, RME, endosomal sorting and exocytosis.

RME is the process of targeted (i.e., receptor-mediated) uptake of macromolecules from the extracellular space into intracellular vesicles to reach endosomes. The RMT receptors at the BBB predominantly internalize through the clathrin-mediated endocytosis (CME) process (Liu AP. et al., 2010; Mayle et al., 2012; Villaseñor et al., 2017; Haqqani et al., 2018a), although caveolin-mediated endocytosis (CavE), and caveolin- and clathrin-independent endocytosis (CIE) have also been implicated for some receptors (Fagerholm et al., 2009; Xin et al., 2011; Ben-Zvi et al., 2014; Haqqani et al., 2022). CME itself is a multi-step process that starts with receptor activation, formation of clathrin-coated pits and recruitment of a large endocytic protein machinery. This leads to budding of the pits (containing both the receptor and ligand) into endocytic vesicles, which then undergo un-coating and fuse with early endosomes to release their contents (Conner and Schmid, 2003).

The second step of the RMT pathway is endosomal protein sorting where decisions are made regarding the fates of the receptor and bound ligand. Several markers have been used to study trafficking of antibodies in various endosomes, including early endosomes (e.g., RAB5A, EEA1), late endosomes/lysosomes (e.g., RAB7A, LAMP1, LAMP2), multivesicular bodies (e.g., RAB27A, CD82, PDCD6IP). In early endosomes, it is decided whether the antibody will recycle back to the luminal side, be degraded, or undergo exocytosis at the abluminal side. Molecules that do not need to be degraded are concentrated in a network of tubular early endosomal subdomains forming sorting tubules, which are destined for the luminal membrane, multivesicular bodies (MVBs) or the trans-Golgi network (TGN), thereby avoiding lysosomal degradation (Maxfield and McGraw, 2004; Grant and Donaldson, 2009). Typically, recycling vesicles recycle the RMT receptor (with or without the ligand) back to the luminal side, whereas the multivesicular bodies (MVBs) receive cargo for degradation or exocytosis. For degradation, the cargo is sent to late endosomes and lysosomes.

The final step of the RMT pathway is the externalization of the receptor-ligand complex to the basolateral side of the barrier. This process is the least understood of the RMT steps. Exocytosis may occur through multiple routes from the early endosomes: directly from vesicles (e.g., sorting tubules), via TGN, or via a direct fusion of MVBs with the abluminal membrane [reviewed elsewhere (Haqqani et al., 2022)].

To date about a dozen RMT receptors have been discovered by involvement in one or more aspects of the RMT process. Examples of these include Tf receptor (TfR) (Pardridge et al., 1991; Yu et al., 2011; Niewoehner et al., 2014; Yu et al., 2014), LDL receptor (LDLR) (Goldstein and Brown, 2009) and LDLR-related proteins (LRP1 and LRP8) (Herz and Strickland, 2001; Xin et al., 2011), INS receptor (INSR) (Coloma et al., 2000; Boado et al., 2010), IGF1 and IGF2 receptors (IGF1R, IGF2R) (Ribecco-Lutkiewicz et al., 2018; Alata et al., 2022; Shin et al., 2022; Yogi et al., 2022), TMEM30A/ATPase complex (Abulrob et al., 2005; Farrington et al., 2014; Webster et al., 2016), and neutral amino-acid transporter CD98 heavy chain (SLC3A2) (Zuchero et al., 2016; Chew et al., 2023; Pornnoppadol et al., 2023). These RMT receptors are described below with more details to identify key criteria, besides involvement in the RMT process, that are important for developing BBB carriers against these receptors.

## 3 Types of transporters and their RMT properties

### 3.1 Iron transporter: transferrin receptor

Transferrin receptor (TfR, CD71, TFRC) is a cell-surface protein and a main source of cellular import of iron, which is a vital element in several biological processes including oxygen transportation, energy generation/mitochondrial function, as well as DNA synthesis and repair (Laskey et al., 1988; Chua et al., 2007; Torti and Torti, 2020). TfR imports iron via receptor-mediated endocytosis of holo-Tf (i.e., iron-bound Tf) into endosomes (Senyilmaz et al., 2015). Inside specific endosomes, holo-Tf-TfR may directly reach the abluminal surface where holo-Tf is released and unbound TfR recycled back (Duck and Connor, 2016). However, more likely the lower pH of specific endosomes releases iron from holo-Tf, producing apo-Tf (i.e., iron-lacking Tf) bound to TfR. The apo-Tf-TfR complex is then recycled back to the cell surface, where the apo-Tf is released from TfR at a neutral pH (Qian et al., 2002). It should be noted that a homolog to TfR, TfR2 is also able to bind circulating Tf (Kleven et al., 2018). However, since expression of TfR2 is lacking at the BBB and is predominantly at erythrocyte precursors and hepatocytes, only TfR is discussed here.

At the BBB, TfR has been shown to act in a bidirectional RMT manner, transporting holo-Tf from the blood to the brain and the apo-Tf from the brain to the blood side (Skarlatos et al., 1995; Zhang and Pardridge, 2001a). TfR has been detected predominantly on the luminal side of the BBB (Zhang et al., 2015; Hill et al., 2021), co-localizes with the CME pathway (Liu AP. et al., 2010; Mayle et al., 2012; Villaseñor et al., 2017; Haqqani et al., 2018a; Haqqani et al., 2018b), early endosomes (Bien-Ly et al., 2014; Niewoehner et al., 2014; Sade et al., 2014; Haqqani et al., 2018a) and MVBs/exocytosing vesicles (Haqqani et al., 2013; Villaseñor et al., 2017; Haqqani et al., 2018b), and recycles back to luminal membrane likely involving the CavE pathway (Pol et al., 1999; Gagescu et al., 2000; Hansen et al., 2003; Lapierre et al., 2007; Leyt et al., 2007). Given its ability to internalize and shuttle via endosomes between luminal and abluminal membranes of the BBB, William Pardridge’s group introduced the RMT approach for brain drug delivery (Pardridge et al., 1987; Pardridge et al., 1991) and ever since TfR has been extensively targeted to deliver drugs through the BBB and into the brain parenchyma.

The protein sequence and structure of human TfR plays a key role in designing antibodies against it. Human TfR is a single-pass type II membrane protein and has 672 amino acids (89–760 aa) on the extracellular domain (ECD) (UniProt consortium, 2024), a significant binding surface accessible at the luminal membrane of the BBB. However, the protein has one O-linked (Hayes et al., 1992) and three N-linked (Lawrence et al., 1999) glycosylation sites in the ECD which could provide some steric hindrance for antibody binding. In addition, 192 amino acids at the C-terminus of the ECD (569–760 aa) are important for Tf binding (UniProt consortium, 2024) and targeting this region could interfere with iron import into the brain. The remaining 481 amino acids of the ECD region, excluding the transferrin-binding site, are not very conserved between human and rodents, being only 68% similar and 53% identical (Table 1). This has made it difficult to raise species cross-reactive anti-TfR antibodies for translational studies, necessitating the development of transgenic mouse expressing human full-length or ECD of TfR (Yu et al., 2011; Niewoehner et al., 2014; Kariolis et al., 2020) for pre-clinical studies. Nonetheless, several labs have successfully developed anti-TfR antibodies, targeting different ECD regions, with different affinities that can cross the BBB.

**TABLE 1:** Key features of known RMT receptors at the BBB^a^.

RMT receptor	Receptor complexity	Transmembrane domains (TMDs)	Luminal/Abluminal	Largest ECD (amino acids)	N-linked glycosylation sites	Human vs. mouse ECD sequence similarity (%)	Human vs. mouse ECD sequence identity (%)	CNS delivery type	Therapeutic cargo delivered	BBB abundance	BBB specificity
TFRC	Single-pass type II	1	Mostly L	672	3	86	76	Antibodies	Anti-BACE; Anti-Aβ; IDS; neprilysin; ProGRN	Abundant (top 30%)	Low
LDLR	Single-pass type I	1	Unknown	767	5	86	79	Peptides (e.g., Transcend)	IL1R antagonist	Abundant (top 40%)	Low
LRP1	Single-pass type I	1	L/A	4400	52	98	98	APOE; Peptides (e.g., Angiopep2)	Placlitaxel; neurotensin; APOE-coated nanoparticles	Abundant (top 30%)	Moderate
LRP8	Single-pass type I	1	Unknown	784	6	89	86	Antibody; peptide		Moderate (top 50%)	High
TMEM30A	2-TMDs	2	L	255	4	91	87	FC5-based antibodies	mGLUR1; anti-Aβ peptide; dalargin; galanin	Highly abundant (top 10%)	Moderate
INSR	Single-pass type I	1	L	929	18	96	95	Antibodies	IDUA; IDS; HEXA; PPT1; ASM; GLB1	Moderate (top 50%)	Low
IGF1R	Single-pass type I	1	L	195	16	97	97	Antibodies	Neurotensin; Anti-alpha-synuclein	Moderate (top 50%)	Low/Moderate
IGF2R	Single-pass type I	1	Unknown	2264	18	90	82	Antibody	LNP-containing p11 gene	Moderate (<50%)	Low
SLC2A1	12-TMDs	12	L	33	1	88	88	Antibodies Glucose	Glucose-coated nanoparticles for SLC2A1	Highly abundant (top 20%)	High
SLC3A2	Single-pass type II	1	L	425	4	81	71	Antibodies	BACE1	Highly abundant (top 10%)	Moderate
Bsg	Single-pass type I	1	Unknown	186	3	71	51	Antibodies		Abundant (top 30%)	Low
LEPR	Single-pass type I	1	Unknown	818	18	84	76	Peptides	Dendrigraft poly-L-Lysine (DGL) containing plasmid DNA	Moderate (<50%)	Low
ITM2A	Single-pass type II	1	Unknown	190	1	96	96	Antibodies		Highly abundant (top 10%)	High
FCGRT	Single-pass type I	1	Unknown	274	1	76	69	Antibodies		Highly abundant (top 10%)	Low
Ideal	Single-pass	**1**	**L>A**	**>200**	**≤1**	**100**	**100**	Antibody	Multiple	Highly abundant (top 10%)	High

^a^ BBB specificity was determined by examining the levels of the RMT receptor in the brain and peripheral tissues. Receptors showing 1.5-fold higher levels in the brain were considered “High,” those showing 1.5-fold higher levels in the periphery were considered “Low” and the values in-between were considered “Moderate.”

Bold values represent desirable features of an ideal RMT receptor.

Numerous anti-TfR antibodies against human, non-human primates (NHPs) and rodent TfR have been engineered to deliver therapeutic cargos across the BBB *in vivo*. Following are few examples of some of the major studies. Watts and coworkers at Genentech generated a bispecific antibody, with anti-TfR BBB-carrier on one arm and anti-β-secretase therapeutic on the other arm, and demonstrated that the antibody can be delivered to the brain and effectively reduce the therapeutic target amyloid beta (Aβ) in both the CSF and the brain of rodents and NHPs (Yu et al., 2011; Bien-Ly et al., 2014; Yu et al., 2014). At Roche, Freskgård and co-workers have developed anti-TfR based Brainsshuttle transport system which when coupled to either an anti-Aβ antibody or Aβ-degrading metallopeptidase neprilysin is delivered to the CNS and substantially reduces Aβ levels in CSF and brain parenchyma (Niewoehner et al., 2014; Campos et al., 2020). At NIH, Brody and coworkers demonstrated *in vivo* target engagement of therapeutic single-domain antibodies (sdAbs) against anti-Aβ and anti- P2X7 receptor fused to anti-TfR sdAb (Esparza et al., 2023). Similarly, Rutkowski and co-workers at Ossianix developed shark anti-TfR sdAb, which was able to successfully deliver TrkB neurotrophin receptor agonist antibody to brain parenchyma and completely prevent neuronal cytotoxicity in a mouse model of Parkinson’s disease (Clarke et al., 2022). Recently, Watts and co-workers at Denali have developed several protein transport vehicles (TVs) in which Fc domains are engineered to have TfR binding site. TVs containing iduronate 2-sulfatase (IDS) (Arguello et al., 2022), progranulin (Logan et al., 2021) and anti-β-secretase (Kariolis et al., 2020) were shown to be successfully delivered to the CNS and demonstrated strong pharmacodynamics effects. The success of targeting TfR to deliver therapeutics into the brain has resulted in several anti-TfR antibodies entering Phase 1 clinical trials. These include delivery of anti-Aβ antibody in Alzheimer’s disease (Roche), recombinant IDS protein in Hunter syndrome (separate clinical trials by JCR Pharmaceuticals Co and Denali Therapeutics), and recombinant progranulin in frontotemporal dementia (Denali Therapeutics), all coupled with anti-TfR BBB-carrier function. Promising efficacy and safety results were recently observed in Phase 2/3 clinical trial of JCR Pharmaceuticals’ anti-TfR-coupled IDS protein, which was recently conducted in 28 Japanese patients with Hunter syndrome (Okuyama et al., 2021), and has recently resulted in the first approval of TfR antibody—enzyme fusion protein for facilitated brain delivery.

While TfR can be targeted to deliver cargo in the brain parenchyma, TfR expression is neither specific to BBB nor to brain tissues (Zhang et al., 2020). TfR is abundantly present in brain tissues and microvessels (Zhang et al., 2020), ranking it in the top 30% by gene expression but it is also similarly (or more) abundantly present in the peripheral vessels and tissues (Uhlen et al., 2010; Zhang et al., 2020). The high peripheral expression has a potential to have off-target side-effects. Adverse drug-related events were observed in Phase 2/3 clinical trial of anti-TfR-coupled IDS protein (Okuyama et al., 2021), although it should be noted that most of the TfR antibody-mediated adverse effects appear to be reversible. However, it remains to be mechanistically determined if any of these events were specifically due to off-target effects either at the peripheral tissues or even within the brain in neurons—since TfR expression is not specific to BBB within the human brain and is abundantly expressed in multiple cell types (e.g., neurons, astrocytes). Thus, while anti-TfR antibodies have provided a significant success in demonstrating clinical proof of concept for the RMT pathway for delivering therapeutics into the brain parenchyma in preclinical and clinical studies, it must be noted that the antibodies could potentially have off-site delivery and adverse but reversible effects since TfR is not a BBB-specific RMT receptor.

### 3.2 Lipid transporters

Several membrane proteins are responsible for transporting lipids and lipoproteins intracellularly via the RME/RMT pathway and thus considered as potential RMT receptors. These include the family of LDL receptor (LDLR), including LDLR itself and LDLR-related proteins 1 and 8 (LRP1 and LRP8, respectively), as well as a lipid filppase complex containing TMEM30A. The discovery of LDLR about 50 years ago by Michael Brown and Joseph Goldstein (for which they won the Nobel Prize in 1985) introduced the concepts of RME, receptor recycling, and feedback regulation of receptors (Goldstein and Brown, 2009), which are all central to the process of RMT. They demonstrated that LDLR plays a key role in cholesterol homeostasis by transporting cholesterol-enriched LDL via the CME pathway into cells, which results in feedback regulation of intracellular cholesterol synthesis (Goldstein and Brown, 2009). LRP1, on the other hand, has a wide range of structurally and functionally diverse ligands that it recognizes on the cell surface (Potere et al., 2019). For some ligands, LRP1 transduces their interactions intracellularly via signaling pathways, while for others (e.g., APOE), LRP1 physically translocates the ligands to intracellular compartments (Herz and Strickland, 2001) via CME or CavE pathways (Xin et al., 2011; Haqqani et al., 2022). Like LRP1, LRP8 also plays roles in both cellular signaling and RME. The receptor plays a critical role during development by mediating Reelin signaling, and also functions as a receptor for the transport of APOE. Besides the family of LDLR, the TMEM30A/ATPase complex involvement in various aspects of the RMT pathway and ability to transport a cargo across the BBB has been shown recently. The complex has a flippase activity that transports amino-phospholipids from the outer to the inner leaflet of cellular membranes and ensures the maintenance of asymmetric distribution of phospholipids. Its role as a an RMT receptor was identified after TMEM30A was discovered to be a receptor for FC5 sdAb (Abulrob et al., 2007) that can cross the BBB via the RMT pathway. The receptor has been shown to internalize via the CME pathway, traffic via early endosomes into MVBs, and exocytose likely via recycling vesicles and exosomes [summarized in Haqqani et al. (2022)].

One of the features of the 4 lipid transporters that might make them attractive targets for antibody generation is the accessibility of their ECD. The LDLR family receptors are single-pass type I membrane proteins and have highly accessible ECDs consisting of 767, 4400 and 784 amino acids, respectively (Table 1). TMEM30A, despite having two transmembrane domains, also has a large ECD of 255 amino acids. LRP1 in fact has the largest ECD of all known RMT receptors considered for BBB delivery. In addition, it is the most conserved of the RMT receptors, with 98% sequence similarity and identity between human and mouse LRP1 proteins. However, LRP1 is also known to bind to >100 ligands for its physiological/pathological functions (Potere et al., 2019), making it challenging to develop anti-LRP1 therapies that do not interfere with the binding of these ligands to LRP1. Furthermore, LRP1 is the most heavily glycosylated RMT receptor, with more than 50 glycosylation sites spread across its entire ECD, which may also limit *in vivo* accessibility. The other 3 lipid receptors, LDLR, LRP8 and TMEM30, have moderate-to-high sequence conservation between human and mouse proteins and are moderately glycosylated (Table 1).

Nonetheless, macromolecules targeting these lipid receptors have been developed and demonstrated to cross the BBB and some have been shown to deliver cargo into the brain. For LDLR and LRP1, although evidence of a successful antibody that can target these receptors and cross the BBB is currently lacking, targeting-peptides derived from the natural ligands of the receptors or discovered through phage display technology have been identified as cargo carriers across the BBB. An example is a 12-amino acid peptide, also referred to as Transcend peptide, originating from melanotransferrin (MTf). While having structural homology to Tf, MTf is a natural ligand for LDLR instead of TfR (Demeule et al., 2002). The Transcend peptide was able to deliver a full-length IgG and interleukin-1 receptor antagonist into brain parenchyma as demonstrated by confocal microscopy and a pharmacodynamic effect in a neuropathic pain mouse model (Thom et al., 2019). Another example is a 19-amino acid, LRP1-targeting peptide called Angiopep-2 that was derived from Kunitz domains (Demeule et al., 2008). Angiopep-2 was demonstrated to efficiently deliver the cancer drug Placlitaxel to gliomas (Thomas et al., 2009), and its pharmacodynamic effects were demonstrated as a neurotensin fusion protein (Demeule et al., 2014). Whereas these peptide ligands against LDLR and LRP-1 provided pre-clinical proof of concept that these receptors are “targetable” for brain delivery of payloads, their limitations including short circulation half-life and instability hindered their clinical development. APOE is another natural ligand of LRP1 that has been demonstrated in a number of studies to deliver nanoparticles containing a variety of payloads through the BBB in *in vitro* models and several animal models (summarized in Hartl et al. (2021)). Phage display technology has been used to develop targeting peptides that bind to ECDs of LDLR (André et al., 2020) or LRP1 (Sakamoto et al., 2017; Sakamoto, 2022). These peptides have been demonstrated to effectively cross the BBB in both *in vitro* and *in vivo* conditions, although their abilities to carry a therapeutic along with them has yet to be shown. However, it should be noted that LRP1 may not be predominantly accessible at the luminal membranes of the BBB and has important role for Aβ clearance in the abluminal membranes (Ramanathan et al., 2015; Khan et al., 2021). Similar to LRP1, anti-LRP8 peptide (Benatuil et al., 2018) and anti-LRP8 antibody (Argiriadi et al., 2023) have been shown to cross the BBB but evidence of their ability to carry a therapeutic cargo into the brain is currently lacking. On the other hand, such evidence has been documented for TMEM30A-binding sdAb FC5. This V_H_H to anti-TMEM30 complex has been shown to effectively cross the BBB in both *in vitro* and *in vivo* models (Farrington et al., 2014; Webster et al., 2016; Ribecco-Lutkiewicz et al., 2018; Kang et al., 2021). FC5 has also been demonstrated to effectively carry short therapeutic peptides, including dalargin, galanin and anti-Aβ peptide (Farrington et al., 2014; Stanimirovic et al., 2014; Webster et al., 2016; Kang et al., 2021), as well as larger proteins such as 150 kDa full IgG towards metabotropic glutamate receptor-1 (Stanimirovic et al., 2014; Webster et al., 2016) into CNS in animal models, triggering corresponding therapeutic effects (Farrington et al., 2014; Webster et al., 2016; Kang et al., 2021).

Similar to TfR the expression of lipid transporters is not BBB-specific, with the exception of LRP8. LDLR and LRP1 are abundantly present in human brain tissues and microvessels, ranking in the top 30% by gene expression, and TMEM30A is even more abundantly present in brain tissues and microvessels, ranking in the top 10% (Zhang et al., 2020). However, the expression is similarly abundant in the peripheral human tissues for both LRP1 and TMEM30A and even higher for LDLR [(Uhlen et al., 2010; Zhang et al., 2020) and Table 1]. Thus, off-target effects due to high peripheral expression in various organs may need to be addressed for peptides and antibodies targeting these RMT receptors. In fact, some adverse effects were observed in a first-in-human study in patients with advanced solid tumors, although they appeared to be reversible (Kurzrock et al., 2012). On the other hand, LRP8 has strong CNS specificity, showing 5 to 10-fold higher expression in the CNS tissues than in peripheral tissues (Uhlen et al., 2010; Zhang et al., 2020). However, the abundance of LRP8 is moderate, ranking below the top 40% by overall gene expression, and not as high as some of the other RMT receptors. Thus, even though several lipid transporters have been validated as RMT receptors and demonstrated the ability to deliver cargo into the brain parenchyma, their specificity, accessibility and/or abundance in peripheral tissues may hinder their development towards clinical trials.

### 3.3 Receptors for insulin and insulin-like growth factors

Receptors for insulin (INS), insulin-like growth factor-1 (IGF1) and −2 (IGF2) are cell-surface proteins that mediate metabolic signals and promote growth. The INS receptor (INSR) and IGF1 receptor (IGF1R) are members of the receptor tyrosine kinase family that are activated following high affinity-binding to their own ligands (INS and IGF1, respectively), and with lower affinity to non-cognate ligands IGF1, IGF2 or INS (e.g., INS can also activate IGF1R, and IGF1 and IGF2 can activate INSR) (Mancarella and Scotlandi, 2018). The activated receptors lead to similar intracellular signaling pathways (PI3K and MAPK) but have distinct functional outcomes; INSR signaling regulates glucose uptake and release, as well as the synthesis and storage of carbohydrates, lipids and protein, whereas IGF1R signaling is involved in cell growth and survival control (Kim and Accili, 2002). IGF2R, on the other hand, is unrelated to either INSR or IGF1R, does not bind to either INS or IGF1, and does not transmit signals intracellularly (Mancarella and Scotlandi, 2018). IGF2R instead is a receptor for IGF2 and mannose 6-phosphate (M6P)—a post-translational modification of proteins—and mediates their transport from various cellular compartments to endosomal/lysosomal compartments (Oshima et al., 1988; McCormick et al., 2008).

The 3 receptors (INSR, IGF1R, IGF2R) are involved in various aspects of the RMT process. Both INSR and IGF1R have been shown to internalize via the CME pathway, the non-CME pathway, and the CavE pathway (summarized in Morcavallo et al. (2014)) and sort through early endosomes and MVBs (Hunker et al., 2006; Haqqani et al., 2022), especially when incubated with their antibodies.

IGF2R plays important roles in intracellular trafficking of IGF2 and M6P-modified proteins. For IGF2, IGF2R acts as a signal antagonist that prevents excess IGF2 signaling by removing it from the extracellular space and trafficking it via endosomes to lysosomes for degradation (Goda and Pfeffer, 1988; Johnson et al., 1990; Jadot et al., 1992). For M6P, IGF2R is known to act as a cation-independent receptor, mediating the delivery of newly synthesized M6P-modified lysosomal enzymes from the Golgi complex to lysosomes via the early endosome (Oshima et al., 1988; McCormick et al., 2008). Given their involvement in RMT processes and intracellular trafficking, the 3 receptors (INSR, IGF1R, IGF2R) are attractive targets for drug delivery into the brain. However, it should be noted that the IGF2/M6P transport by IGF2R is progressively lost with aging from neonates to adult, and in fact, by adulthood, the BBB has totally lost this transport capacity (Urayama et al., 2004), suggesting that the IGF2R system might be limited to delivery in neonates only.

The protein sequences and structures of human INSR, IGF1R and IGF2R have several features that make them attractive targets for developing binding antibodies. All 3 receptors are single-pass type I membrane proteins and have highly accessible ECDs consisting of 929, 195 and 2264 amino acids, respectively. In addition, protein sequences for INSR and IGF1R are highly conserved among various species—likely amongst the highest for all known RMT receptors—with each of the human sequences being >95% identical to the sequences of their murine counterpart (Table 1). Human IGF2R, while being only 82% identical to mouse, has a very large ECD and as a result has more conserved residues with mouse (>1850 aa) than the conserved residues of INSR and IGF1R combined. However, the 3 receptors are heavily glycosylated with at least 16 N-linked glycosylation sites in the ECDs, which can potentially provide strong steric hindrance for antibody binding. Furthermore, the receptors have multiple regions throughout their ECDs that are important for their respective ligand binding (Bergman et al., 2013; Ye et al., 2017; Li et al., 2019). Thus, generating antibodies to INSR, IGF1R and IGF2R that do not interfere with their physiological functions could be challenging and evidence needs to be provided that these antibodies do not interfere with downstream pathways.

Antibodies to INSR, IGF1R and IGF2R have been developed and shown to deliver cargo across the BBB *in vivo*. The most well characterized anti-INSR antibody is the humanized monoclonal antibody called HIRMAb (Pardridge, 2017) that binds an epitope within the 469–592 amino acid region of the receptor (Pardridge et al., 1995). The antibody has been shown to successfully carry a number of enzymes as cargo across the BBB, including α-l-iduronidase (IDUA) (Pardridge et al., 2018), IDS (Boado et al., 2014), hexosaminidase A (Boado et al., 2019), palmitoylthioesterase-1 (Boado et al., 2019), acid sphingomyelinase (Boado et al., 2019) and beta galactosidase-1 (Boado et al., 2019). The HIRMAb-IDUA antibody has shown positive results in clinical studies (Pardridge et al., 2018) and is currently in clinical trials, although some mild adverse effects including infusion related reactions and transient hypoglycemia have been observed in pediatric patients (Giugliani et al., 2018). Anti-IGF1R antibodies have been shown also to cross the BBB and carry cargos into the brain parenchyma. These include multi-species cross-reactive, V_H_H sdAbs IGF1R3, IGF1R4 and IGF1R5 that can successfully carry cargo of a wide range of molecular weight sizes (<10 kDa–140 kDa) across the BBB either as sdAbs or in Fc format. The proof of concept for use of these IGF1R V_H_Hs has been obtained in human and rat *in vitro* BBB models, as well as in rat, mouse and NHP *in vivo* models (Ribecco-Lutkiewicz et al., 2018; Sheff et al., 2021; Alata et al., 2022; Yogi et al., 2022). These sdAbs target receptor epitopes that do not interfere with the physiological functions (Sheff et al., 2021; Sheff et al., 2023). Recently, ABL Bio Inc. demonstrated that an anti-IGF1R scFv (Grabody B) fused with therapeutic anti-alpha-synuclein IgG crosses the BBB and improves the neuropathology and behavior in a Parkinson’s disease animal model better than the therapeutic IgG alone (Shin et al., 2022). Finally, an anti-IGF2R antibody called IGF-II was demonstrated to carry lipid nanoparticles-containing p11 gene and deliver it in rat and mouse brain parenchyma (Gandhi et al., 2019), although details of the antibody and *in vitro* BBB-crossing are unspecified, especially the fact that transport capacity of IGF2R has been shown to be lost in adulthood (Urayama et al., 2004).

Given the ubiquitous role of insulin and related growth factors, their receptors are not uniquely expressed at the CNS. INSR and IGF2R (mostly neuronal) are moderately expressed in human brain tissues and microvessels, and their expression is actually higher in many of the peripheral human tissues (Uhlen et al., 2010; Zhang et al., 2020). Similar is seen for IGF1R, although the brain expression is slightly higher than peripheral tissues (Zhang et al., 2020) and it is easily accessible on the luminal surface of the BBB (Hill et al., 2021). Nonetheless, while INSR and related receptors have been demonstrated to deliver cargo successfully through the BBB and into the CNS, their roles in essential physiological functions are potential liability and requires careful engineering of binding antibodies to mitigate interference with these functions.

### 3.4 Solute carrier transporters

Solute carriers (SLCs) represent a major superfamily of membrane transporters with more than 450 members classified into 66 families (Hu et al., 2020). These proteins mediate the exchange of substances including ions, nutrients, signaling molecules and drugs across biological barriers. Glucose transporter 1 (Glut1) is a member of the SLC2 family (SLC2A1) that provides basal metabolic energy to the cells mainly in the form of glucose although it can also transport galactose, mannose and glucosamine with different efficiencies (Calvo et al., 2010). The transport of sugars by this transporter is bidirectional with passive diffusion solely based on a concentration gradient. The heavy chain of the cluster of differentiation 98 (CD98hc) is a member of the SLC3 family (SLC3A2) forming heterodimeric amino acid transporters by interacting covalently with one of six light chain subunits from the SLC7 family. In the heterodimer, the heavy subunit is responsible for the trafficking of the transporter to the plasma membrane while the light subunit catalyzes the transporter function (Fotiadis et al., 2013), conferring substrate specificity to the molecule. In addition, CD98hc interacts with the cytoplasmic tails of β-integrin subunits and participates in cellular signaling thereby promoting cellular migration, survival, spreading and growth (Zent et al., 2000; Cantor et al., 2008).

Both CD98 and Glut1 have been shown to internalize via CIE pathways (Eyster et al., 2009) entering the cells independently of dynamin, but dependent upon plasma membrane cholesterol and accumulating in vacuoles containing an active form of the GTPase Arf6 (Arf6Q67L). However, the two proteins employ divergent itineraries to tubular recycling endosomes. Once inside the cells, Glut1 was found to merge with endosomes containing Tf and early endosomal antigen 1 (EEA1) before entering the recycling tubules; in contrast, CD98 utilizes novel downstream sorting mechanisms and directly enters the recycling tubules by-passing the EEA1-positive compartment (Eyster et al., 2009). CD98 likely utilizes the CLIC/GEEC internalization pathway since it is a GPI-anchored protein.

The structure of Glut1 is extremely complex with 12 trans-membrane (TM) domains and very limited sequences available at the cell surface for the generation of antibodies. Only the first extra-cellular loop (ECL) containing 33 amino acids offers sufficient space for antibody targeting whereas the other five loops are likely too small. ECL1 contains a single N-linked glycosylation site and interestingly, the pattern of glycosylation has been shown to vary depending on the localization of the transporter (Patching, 2017). The glycosylated form of Glut1 is observed at the BBB and on the membrane of erythrocytes whereas the low molecular weight non-glycosylated form can be found at the cell membrane of astrocytes and to some extent microglia. The sequence of Glut1 is highly conserved between species with 97% identity between human and mouse for the full-length protein and 88% identity for ECL1 (Table 1). Finally, the binding sites for glucose are found within TM domains (Deng et al., 2014) and therefore, the targeting of any ECL with antibodies is unlikely to affect the physiological function of the transporter. In contrast, CD98hc is a single-pass type II membrane N-glycoprotein composed of an intracellular N-terminal domain, a single transmembrane domain and a large C-terminal ECD (425 aa) which is widely accessible for the generation of antibodies (Table 1). However, this domain only shares 71% identity with the murine protein combined with an inconsistent glycosylation pattern and notable structural differences (Deuschle et al., 2019). Therefore, the development of cross-reactive antibodies with therapeutically significant affinities against this protein is likely to pose a challenge. In addition, cryo-EM analysis of the ECD revealed the presence of four glycans with the potential to sterically hinder antibody binding (Lee et al., 2019).

Given the very high energy demand of the brain and the fact that glucose is the main source of energy for this sophisticated organ, it is no surprise that the Glut1 receptor is remarkably abundant at the BBB compared to many other receptors and transporters (Uchida et al., 2011). Taking advantage of the very high level of expression of this protein in BECs, several nanocarriers were developed in which glucose was used as the targeting ligand decorating the surface of the delivery vessels (Qin et al., 2010; Xie et al., 2012). Nevertheless, although these earlier versions of the system demonstrated cellular uptake and BBB crossing *in vitro*, their analysis *in vivo* only led to a modest accumulation of the nanocarriers in the brain (less than 1% dose/g-brain) following intravenous injection in mice. The first real success of using glucose-modified nanoparticles was obtained under hypoglycemic conditions (Anraku et al., 2017), which have been shown to result in a significant increase of the luminal expression of Glut1 (Simpson et al., 1999) therefore facilitating target engagement by blood-borne vehicles and leading to a significant increase in transcytosis upon addition of glucose. When fasting mice received a glucose solution, accumulation of glucose-modified nanocarriers at high levels in the brain (∼6% dose/g-brain) was observed. This glycemic control strategy was later applied to deliver nanoparticles containing antisense oligonucleotide (ASO) for brain gene knockdown (Min et al., 2020) or antibody fragments for Alzheimer’s disease treatment (Xie et al., 2020) in the brain. In another set of experiments, Glut1 together with CD98hc were both selected from a proteomic screen aiming at identifying transmembrane proteins highly expressed in isolated mouse primary BECs (Zuchero et al., 2016). Subsequent generation of three antibodies targeting Glut1 (one antibody) or CD98hc (two antibodies) demonstrated a significant accumulation in the brain *in vivo*. In particular, systemic administration of CD98hc antibodies showed the highest brain concentrations of all RMT targets investigated. Reformatting of anti-CD98hc antibodies into bi-specific molecules targeting CD98hc on one arm and the β-site amyloid precursor protein cleaving enzyme 1 (BACE1) on the other arm led to the reduction of brain Aβ by 30%–40% confirming the penetration of antibody molecules into the brain parenchyma. Importantly, targeting of CD98hc by antibodies did not affect the total brain expression level or subcellular localization of the transporter nor did it affect the level of amino acid transport. Delivery into the brain of an anti-CD98hc antibody was also demonstrated in non-human primates (Edavettal et al., 2022). Finally, a mouse specific scFv fused to the C-terminus of one heavy chain of an IgG molecule was shown to cross the BBB and engage several endogenous targets in the brain parenchyma of wild-type mice (Pornnoppadol et al., 2023). Notably, the CD98hc shuttle demonstrated sustained brain concentration up to 1 week following administration. Although experiments with these transporters have been limited to mice and non-human primates, proteomics on human brain microvasculature revealed a high expression of both CD98hc and Glut1 (Uchida et al., 2011) suggesting that both targets hold the potential to translate into humans. In fact, a human/mouse cross-reactive transport vehicle composed of an anti-CD98hc IgG and a modified Fc region was recently shown to reach the brain in humanized CD98-knock in mice (Chew et al., 2023) with slower and more prolonged kinetic properties compared to other BBB shuttles and no safety issues noted.

Both Glut1 and Cd98hc are amongst the most abundant proteins in brain microvessels (Zuchero et al., 2016; Zhang et al., 2020). While Glut1 is expressed in virtually all tissues, it appears to be selectively enriched in brain microvessels compared to peripheral tissues and total brain (Zhang et al., 2020) making it quite attractive as a brain delivery target. In contrast, in addition to the high expression observed in brain microvessels, high levels of the CD98hc transporter have been detected in the lungs and the spleen as well as in total brain posing a significant risk for off-target side effects upon targeting of this protein although nothing has been reported to date.

### 3.5 Emerging RMT targets

Although the delivery of cargo across the BBB using known RMT receptors described in the previous sections has demonstrated some success, some important limitations remain and no receptor investigated to date is optimal. In recent years, several groups have invested efforts to attempt to identify novel RMT targets with ideal delivery properties. Notably, selective and enriched expression at the BBB compared to whole brain and periphery has been the critical criterion.

Through a screen for proteins highly expressed in mouse BECs leading to the identification of Glut1 and CD98hc as described in the sections above, Zuchero et al. (2016) also identified basigin as a potential RMT target. This protein is a single-pass type I transmembrane protein belonging to the immunoglobulin (Ig) superfamily containing an ECD of 186 amino acids, a single-chain transmembrane domain and a short cytoplasmic tail. The ECD of the most common basigin isoform contains two Ig-like domains covalently inter-linked (Yu et al., 2008) and three N-linked glycosylation sites which are essential for the cell membrane localization of the protein (Huang et al., 2013) but that could sterically interfere with the binding of antibodies. Moreover, this domain shares only 51% identity with the mouse basigin ECD severely impeding the development of cross-species reactive anti-basigin therapies (Table 1). Basigin acts as a co-receptor for the lactate transporter, monocarboxylate transporter 1 (MCT1) mediating its cell surface translocation which is essential for the function of the transporter (Kirk et al., 2000). Basigin has also been shown to interact either directly or indirectly with several other partner molecules and is involved in numerous physiological and pathological processes (Muramatsu, 2016) making it difficult to develop basigin-targeting antibodies that do not interfere with the function of the receptor. Although the expression of basigin is not exclusive to the brain, it appears to be significantly enriched in BECs compared to other organs (Tam et al., 2012; Zuchero et al., 2016; He et al., 2018). However, this protein is also highly expressed in all leukocytes (Weidle et al., 2010), erythrocytes (Coste et al., 2001) and platelets (Schmidt et al., 2008) in which it plays a critical role and dysregulation of its expression has been associated with an array of immunological diseases (Hahn et al., 2015). Therefore, the targeting of this receptor for BBB crossing presents significant risks of having detrimental consequences on immune cell development and function. Nevertheless, following identification of high levels of basigin expression in mouse BECs, two antibodies against this protein were generated followed by analysis of *in vivo* binding after IV administration in mice (Zuchero et al., 2016). Interestingly, while the two anti-basigin antibodies accumulated in the brain, the high affinity antibody was found at levels similar to an anti-TfR control whereas only modest accumulation was observed for the lower affinity antibody against Basigin. This is consistent with the affinity-dependent differences obtained with anti-TfR antibodies at trace dose levels (Yu et al., 2011). In contrast, both basigin-targeting antibodies were found to accumulate at similar levels comparable to the anti-TfR control antibody following injection with higher therapeutic doses (Zuchero et al., 2016) and all antibodies were able to reach the brain parenchyma as demonstrated by microvessel depletion techniques. In view of better understanding the RMT properties of basigin, a larger panel of antibodies were generated targeting different epitopes of the receptor ECD with variable affinities (Christensen et al., 2021). Antibodies from all epitope bins demonstrated the ability to bind and internalize to variable levels in a human brain endothelial cell line expressing basigin. Overall, the differences in the binding characteristics observed within each epitope bin did not correlate with differences in affinities although this remains to be further confirmed. In a subsequent study (Christensen et al., 2021), the same group investigated the transcytosis ability of selected antibodies in a porcine *in vitro* BBB model and found that all antibodies were able to cross the BBB endothelial cell layer to then internalize in co-cultured astrocytes. The analysis of the trafficking pathway in porcine BECs revealed a CavE-independent internalization mechanism followed by the accumulation of anti-basigin antibodies in EEA1 and TfR-containing recycling vesicles and no localization with late endosomes or lysosomes.

The integral membrane protein 2A (ITM2A) is a single-pass type II integral membrane protein composed of four distinct regions which are the hydrophobic, the linker, the extracellular BRICHOS and the intracellular C-terminal domains. The ECD (190 aa) is of particular interest since it is large and exposed at the cell surface available for antibody targeting and it shares more than 95% homology with the mouse protein (Table 1). ITM2A was first identified as a marker for chondro-osteogenic differentiation (Deleersnijder et al., 1996) whose role was later confirmed by others (Tuckermann et al., 2000; Van den Plas and Merregaert, 2004; Boeuf et al., 2009). Since then, it has also been shown to play a role in myogenic differentiation (Van den Plas and Merregaert, 2004; Lagha et al., 2013) and in the regulation of autophagy (Namkoong et al., 2015; Zhou et al., 2019). However, little is known about its biological function in the brain. Several studies identified ITM2A as a brain endothelial cells-specific protein with low expression in other brain cell types (Zhang et al., 2016; McKenzie et al., 2018; Hu et al., 2020) including astrocytes, oligodendrocytes, neurons and microglia and in peripheral organs (Li et al., 2002; Pardridge, 2007; Bangsow et al., 2008; Feng et al., 2019; Hu et al., 2020) such as liver, kidney, heart and lung. Nevertheless, the involvement of this protein in RMT pathway remains unclear. More recently, Cegarra et al. (2022) at Sanofi interrogated the use of this protein as a delivery agent across the BBB. Antibodies targeting extracellular regions of the protein were generated and their ability to bind and internalize in cells overexpressing ITM2A was demonstrated. However, attempts made by the authors to demonstrate BBB crossing capabilities of ITM2A-specific antibodies *in vitro* or *in vivo* were inconclusive. While the overall characteristics of ITM2A point to a good RMT target, its validation for brain delivery remains to be shown.

Leptin, a neuropeptide involved in the regulation of appetite is secreted by peripheral adipocytes and acts centrally on leptin receptor-expressing brain cells to reduce food intake and retard weight gain (Houseknecht et al., 1998). Evidence suggests that this peptide reaches the brain parenchyma through its receptor following clathrin- and calveolae-mediated energy dependent endocytosis (Banks et al., 1996; Barr et al., 1999) although other mechanisms of entry might exist (Banks et al., 2002; Banks, 2004). The leptin receptor (LERP) is a single-pass type I membrane protein which is expressed in several regions of the brain including the choroid plexus (Tartaglia et al., 1995) and the BBB (Golden et al., 1997) in addition to many other peripheral organs and tissues with enrichment in the lungs (Zhang et al., 2020). The non-CNS specificity of this receptor could pose a significant challenge to the development of brain delivery vectors. The extracellular part of the receptor (818 aa), although widely accessible for antibody targeting in terms of size, contains several complex structural domains from which the second cytokine receptor homology (CRH2) module has been identified as the high-affinity binding domain for leptin (Iserentant et al., 2005). The targeting of this domain therefore represents a risk to interfere with the physiological function of the protein. In addition, the extracellular part of LERP has limited identity with the mouse domain (76%) and is highly glycosylated representing a major obstacle to the generation of antibodies (Table 1). In a search for regions responsible for brain uptake, peptides covering the full-length human leptin protein were synthesized and assessed for their ability to reach the brain parenchyma *in vivo* in rats (Barrett et al., 2009). Two peptides (positions 1–33 and 61–90) demonstrated higher brain uptake compared to control molecules at levels that were similar to those of leptin. Further dissection of peptide 1–33 led to the identification of residues 12–33 with increased brain uptake whereas truncation of the 61–90 peptide led to significantly reduced brain accumulation. The peptides were shown to reach the brain parenchyma through a saturable mechanism. Interestingly, the two regions covered by the peptides were found to be involved in the binding to the leptin receptor confirming the dependency of this interaction for brain uptake. In a subsequent study (Liu Y. et al., 2010), the most efficient peptide (leptin30) was further modified by covalent linkage at the surface of synthetic polymers leading to efficient brain-targeted gene delivery both *in vitro* and *in vivo*.

Finally, the neonatal crystallizable fragment receptor (FcRn) is a single-pass type I membrane glycoprotein forming heterodimers composed of a major histocompatibility complex Class-I like heavy chain and a non-covalently associated β2-microglobulin (β2m) light chain. The heavy chain consists of three structural ECDs (α1, α2 and α3), one transmembrane domain and a short cytoplasmic tail. The overall extracellular region of the heavy subunit is composed of 274 amino acids sharing 69% identity with its mouse counterpart which could limit the generation of cross-species reactive molecules (Table 1). In addition, the ligand binding sites have been mapped to residues within the α1 and α2 ECDs (Giragossian et al., 2013) thereby limiting accessible epitopes for antibody targeting without altering the physiological function of the receptor. FcRn interacts with the fragment crystallizable Fc domain of immunoglobulin G (IgG) molecules and with albumin to regulate their bi-directional transport across numerous biological barriers including the epithelia found at the placenta (Story et al., 1994; Leach et al., 1996), the lungs (Spiekermann et al., 2002), the kidneys (McCarthy et al., 2000; Kobayashi et al., 2002) and the intestines (Rodewald, 1976; Simister and Mostov, 1989) translating in a ubiquitous expression across organs and tissues potentially implying off-target sides effects observed with anti-FcRn therapies. The interaction of IgGs and albumin with FcRn is responsible for their prolonged plasma half-life creating a protein reservoir that is protected from lysosomal degradation and subsequently recycled to the extracellular space (Lencer and Blumberg, 2005). At the BBB, FcRn has been shown to regulate the efflux of antibodies from the brain parenchyma into the blood following reverse transcytosis (Zhang and Pardridge, 2001b; Cooper et al., 2013). Recently, Tien et al. (2023) from Teva proposed that under the appropriate conditions, the receptor could allow for the influx of antibodies from the blood capillaries into the brain and suggested FcRn as a potential RMT target for the development of brain-targeting therapeutics. In this study, three amino acid substitutions (YTE) known to improve the affinity of antibodies for the mouse FcRn at acidic and neutral pH (6.0 and 7.4, respectively) (Dall’Acqua et al., 2002) were introduced within antibody Fc domains resulting in a strong accumulation in the brain parenchyma for an extended period of time (504 h) following IV administration in mice. This was in contrast to antibodies bearing the WT Fc domain for which only very low levels could be detected in the mouse brain. Importantly, the improved translocation of YTE-modified antibodies was independent of their target antigen or functionality while it depended on their interaction with the FcRn receptor leading to cellular internalization and trafficking. Although the experiments were exclusively conducted in mice, the level of FcRn expression in endothelial cells is comparable between human and mouse (Latvala et al., 2017) and this strategy is predicted to be clinically relevant. However, the mutations that were applied in this study do not translate in the same effect in humans than in mice and are unlikely to improve BBB transport in humans. While the YTE substitutions resulted in an improved target engagement at both pH 6.0 and pH 7.4, in humans the same mutations increase the binding at pH 6.0 but not pH 7.4, thereby enhancing FcRn-mediated antibody recycling from endosomes and increasing antibody half-life (Dall’Acqua et al., 2002). Specific mutations that would enhance the interaction of antibodies with the FcRn receptor in a pH-independent manner in humans would have to be independently identified and validated before the real nature of this receptor as a clinically-relevant RMT target for brain delivery can be established. Additionally, it is not clear why these engineered antibodies would behave differently in BECs compared to peripheral ECs and this approach is likely to result in a non-selective increase of transcytosis in all tissues of the body leading to important safety issues.

In addition to the candidates described above, other proteins emerged from early receptor identification studies where in most cases the mechanism of their potential BBB crossing remains unclear. These include the intracellular adhesion molecule 1 (ICAM-1) and podocalyxin (PODXL) which were identified as potential RMT targets from analyses of BBB abundant cell-surface proteins with endocytic properties (Agarwal et al., 2010; Ito et al., 2020) ICAM-1 is however expressed in several cell types of multiple organs with no real enrichment in the brain and has been investigated more as a target for diseases with a wide range distribution such as lysosomal storage disorders (Hsu et al., 2012; Muro, 2012; Solomon et al., 2022) although ICAM-1-targeted carriers have shown the ability to reach the brain amongst other organs (Papademetriou IT. et al., 2013; Papademetriou J. et al., 2013; Hsu et al., 2014) with the highest accumulation observed in the lungs. While PODXL was found to be selectively expressed and to internalize in MBECs (Agarwal et al., 2010; Ito et al., 2020), further studies are required to elucidate its role in the brain and its function as an RMT target. Transthyretin (Ttr) was identified by screening the rat serum proteome for candidates with the ability to transmigrate in an *in vitro* rat BBB model (Kim et al., 2015). When conjugated at the surface of Quantum Dot (QD) nanoparticles, Ttr-targeted particles demonstrated the ability to cross the BBB both *in vitro* and *in vivo*. Although the distribution of Ttr-QDs in the brain was significantly increased compared to control molecules, a strong accumulation was also observed in the liver and to lower extent in the kidney, lung and colon suggesting that Ttr may not represent a safe alternative to current RMT targets.

## 4 Key criteria for an RMT receptor

At least a dozen RMT receptors have been discovered at the BBB and have been targeted for delivery of therapeutics into the brain (Table 1). While they all have different desirable features that have led to their successes in preclinical and even clinical studies, they also have different limitations and concerns that need to be addressed on the path to clinical translation. So far, none of the known RMT receptors have all the desirable features; therefore, further explorations of the BBB selective “transporter” proteome are currently under way, with novel targets expected to be discovered (or publicly disclosed).

Hence, here we list some of the key criteria for identifying an “ideal” RMT receptor.

1. *Role in transport.* Beside their involvement in various RMT processes, a key biological function that is common amongst most (if not all) of the known RMT receptors is that they are involved in cellular trafficking or transport of natural ligands and nutrients, including transport of growth factors, iron, lipid, glucose and other molecules across the BBB. Thus, when choosing a receptor, it might be important to favor those that are naturally involved in transport and understand its endocytic pathways and intracellular trafficking routes (Haqqani et al., 2018a; Haqqani et al., 2018b). However, as a caveat, antibodies or peptides that target these receptors must not interfere with this physiological function.
2. *Abundant expression in brain microvessels.* High expression of receptors in brain microvessels is essential for effective therapeutic delivery to the brain. Expression can be determined from omic profiles (e.g., RNAseq, transcriptomics) of brain microvessels and ranking all the genes by abundance (e.g., top 20% as “highly abundant,” 20%–40% as “abundant,” 40%–60% as “moderate,” and <60% as “low abundant”). Receptors with “low” or no brain vascular expression are discouraged since antibodies or peptides targeting them might not be delivered into the brain to achieve pharmacological effect.
3. *BBB selective.* Beside abundant expression in brain microvessels, low or no expression in peripheral tissues (e.g., lung, liver, kidney) as well as other regions of the brain (e.g., neurons) is important. This BBB selectivity is needed to ensure minimal off-site delivery and adverse side-effects of the therapeutic.
4. *Receptor accessibility.* Most of the known RMT receptor-targeting antibodies and peptides are administered systemically, usually in the blood (via intravenous injection). Thus, unhindered access to the RMT receptor is necessary for the antibodies or peptides to easily go from the blood to the brain side. Several key criteria for *receptor accessibility* have been mentioned throughout this review. These include: a) availability of the receptors on the luminal membranes (blood-facing side) of endothelial cells in brain microvessels; b) presence of a large ECD (ideally >100 amino acids) that has minimal hindrance from post-translational modifications (e.g., glycosylation); c) having a single transmembrane domain (TMD), which has shown more success in brain delivery than multi-pass membrane proteins; d) minimal interference with natural ligands such that biological function would not be affected by targeting antibodies.
5. *Translatable.* Sequence conservation of the ECD is another key criterion that should be considered when deciding on an RMT receptor in order for it to be translatable among multiple species. For receptors that are not highly conserved between human and rodents (e.g., TfR), it is difficult to make species cross-reactive antibodies or peptides to translate studies into pre-clinical settings, and instead transgenic models expressing human full-length or ECD of the receptors are required to study them in rodent models (Yu et al., 2011; Niewoehner et al., 2014; Kariolis et al., 2020). It is thus important to have high sequence similarity/identity (>95%) between human and rodent sequences.

## 5 Conclusion

The concept of receptor-mediated transcytosis as a pathway for brain delivery of biological therapies, most notably antibodies and therapeutic proteins, has made a significant evolution in the last decade, driven by both better mechanistic understanding of trafficking pathways within endothelial cells that can result in exocytosis, as well as by the development of receptor-binding ligands that allowed testing and confirmation, or revision, of the concept in preclinical *in vitro* and *in vivo* models, and most recently, in clinical studies. Whereas the studies with the “classical” target, TfR, that have been initiated in early 90s, have recently resulted in the first approval of TfR antibody—enzyme fusion protein for facilitated brain delivery—and long-awaited clinical proof of concept for RMT as a valid pathway to exploit for brain delivery of biologics, the recent advances have been driven by a search for other RMT receptors using advanced sequencing and proteomic techniques. The rapid generation of antibody tools to validate the function of these receptors provided some critical insights—firstly that they “belong” to vastly distinct functional classes of molecules, including tyrosine kinase receptors, growth factor and neuropeptide receptors, lipid transporters, and even solute carrier family transporters. The ability of these diverse receptors to shuttle antibody/protein cargo across the BBB, demonstrated in various preclinical model systems, vastly expanded horizons of RMT research and open possibilities to discover better and more efficacious transporters, or those more appropriate for particular types of therapeutic cargos. With discovery of each of these “non-canonical” RMTs, comes further understanding of the mechanisms of their internalization, endosomal sorting and cargo “hand-over,” as well as their function in general. Translating from the initial discovery/proof of mechanism studies into development and clinic is arduous and a long process and many of these nominated novel RMTs may fail along the path; however, the optimism and drive in the field to further exploit this mechanism for design of better neurotherapeutics is high and bolstered by initial clinical demonstrations of successes of the first, prototypical TfR that followed through this path.
